# Effect of six hand operation nursing applied in microscopic apical surgery

**DOI:** 10.1590/1980-220X-REEUSP-2024-0383en

**Published:** 2025-05-09

**Authors:** Yahua Liu, Wei Zhang, Su Jiang, Jie Zhang, Li Dai

**Affiliations:** 1 Nanjing University, Medical School, Institute of Stomatology, Nanjing Stomatological Hospital, Department of Endodontics, 210000, Nanjing, China.

**Keywords:** Periapical Tissue, Infections, Microscopy, Nursing, General Surgery, Tecido Periapical, Infecções, Microscopia, Enfermagem, Cirurgia Geral

## Abstract

**Objective:**

To investigate the effect of six hand operation nursing applied in microscopic apical surgery.

**Method:**

Subjects (90 in total) were recruited from patients undergoing microscopic apical surgery in our hospital between March 2022 and March 2024, and assigned to a control group (n = 45) and an observation group (n = 45) using a random number table.

**Results:**

The overall response rate was higher in the observation group than that in the control group (P < 0.05). The levels of tumor necrosis factor-α and interleukin-6 at 3 d after nursing dropped in the two groups in comparison to those before nursing, and they were lower in the observation group than those in the control group (P < 0.05). The overall satisfaction with nursing rose in the observation group in comparison with that of the control group (P < 0.05).

**Conclusion:**

Six hand operation nursing applied in microscopic apical surgery can shorten operation duration, reducing the risk for infections.

## RESUMO

**Objetivo:**

Nosso objetivo foi investigar o efeito de seis mãos de enfermagem em operações manuais aplicada em cirurgia apical microscópica.

**Método:**

Os indivíduos (90 no total) foram pacientes recrutados submetidos à cirurgia apical microscópica em nosso hospital entre março de 2022 e março de 2024. Os participantes foram designados para um grupo controle (n = 45) e um grupo observação (n = 45) usando uma tabela de números aleatórios.

**Resultados:**

A taxa de resposta geral foi maior no grupo observação do que no grupo controle (P < 0,05). Os níveis de fator de necrose tumoral-α e interleucina-6 em 3 dias após a enfermagem caíram nos dois grupos em comparação com aqueles antes da enfermagem, e foram menores no grupo observação do que aqueles no grupo controle (P < 0,05). A satisfação geral com a enfermagem aumentou no grupo observação em comparação com a do grupo controle (P < 0,05).

**Conclusão:**

A enfermagem em seis operações manuais aplicada em cirurgia apical microscópica pode encurtar a duração da operação e reduzir o risco de infecções.

## INTRODUCTION

Periodontitis has a gradually increasing prevalence rate and significantly impacts individuals’ daily lives^(1)^. Currently, root canal therapy is the primary treatment for patients with periodontitis who do not respond to conservative approaches. Although both root canal therapy and re-treatment have shown considerable advancements in therapeutic efficacy, residual apical lesions in affected teeth are still observed in some patients, severely impairing their quality of life^(2)^.

In most cases, periapical lesions are managed through apical surgery, which aims to eliminate periapical tissue infections while promoting tissue regeneration and rapid healing^(3)^. This procedure is typically conducted in an outpatient setting, where maintaining aseptic conditions can be challenging. Additionally, a significant bacterial load in patients’ saliva poses a risk of contaminating surgical instruments and medical personnel’s hands, potentially leading to cross-infections and hindering postoperative recovery^(4)^. Therefore, effective infection control during apical surgery is of paramount importance.

Apical surgery is a crucial method for removing infected root tip tissues and facilitating tissue healing. In recent years, the success rate of this procedure has significantly improved with the incorporation of surgical microscopes^(5,6)^. Microscopic techniques provide an enhanced surgical field of vision, enabling precise identification of periapical physiological structures. However, microscopic surgery imposes high demands on both surgeons and nursing staff. Strict adherence to sterile techniques is essential, and surgical maneuvers should be minimized to the greatest extent possible^(7)^.

In conventional nursing care, a single doctor typically collaborates with one nurse. However, this standard practice does not meet the heightened technical and procedural demands of microscopic surgery^(8,9)^. The efficiency of perioperative nursing cooperation in microscopic procedures is directly related to both the risk of cross-infection and overall surgical outcomes^(10)^. Therefore, effective collaboration plays a crucial role in ensuring rapid postoperative recovery.

To enhance the quality of medical care, a circulating nurse—responsible for maintaining a controlled transition between contaminated and uncontaminated areas—has been introduced into surgical teams, giving rise to the concept of six-hand operation nursing^(11)^. This nursing model, which requires experienced medical personnel, has been shown to reduce the duration of both diagnosis and treatment, making it highly preferred by patients^(12)^.

At present, research on the clinical efficacy of this nursing model in microscopic apical surgery remains limited, particularly concerning its impact on infection control. Given this gap in knowledge, this study enrolled 90 patients who underwent microscopic apical surgery at our hospital over the past two years to assess the effectiveness of six-hand operation nursing. The findings aim to inform future nursing protocols and enhance perioperative care strategies.

## METHOD

### Subjects

The 90 patients undergoing microscopic apical surgery in our hospital from March 2022 to March 2024 were divided into a control group (n = 45) and an observation group (n = 45) using a random number table. The control group was composed of 25 males and 20 females aged 22–51 years old, with (33.25 ± 3.41) years old on average. The lesion diameter of the diseased tooth was 7–13 mm, with a mean of (8.45 ± 1.20) mm. The body mass index was 20–24 kg/m^2^, with an average of (22.36 ± 1.47) kg/m^2^. In the observation group, there were 23 males and 22 females aged 23–51 years old, with a mean of (33.86 ± 3.09) years old. The lesion diameter of the diseased tooth was 7–14 mm, with an average of (8.64 ± 1.17) mm. The body mass index ranged from 21 kg/m^2^ to 24 kg/m^2^, with (22.53 ± 1.37) kg/m^2^ on average. The general data were well-balanced between the two groups (P > 0.05).

## INCLUSION AND EXCLUSION CRITERIA

The undermentioned inclusion criteria were employed: 1) patients with failure in the first root canal treatment and meeting the indications for reoperation, 2) those with unclosed root tip according to X-ray images, 3) those with normal coagulation function, and 4) those who had voluntarily signed the informed consent form.

The exclusion criteria involved: 1) patients with other infectious diseases, 2) those with root fractures in the affected tooth, 3) those with metabolic diseases, 4) those with severe dental pulp lesions, 5) those with lateral perforation or longitudinal fissure of the root canal, 6) those with multiple organ lesions, 7) those with root development malformation, or 8) those with mental disorders.

## METHODS

In the control group, conventional nursing care was implemented, involving one assistant nurse and one surgeon. Specifically, the assistant nurse assisted the surgeon during the procedure by helping patients adjust their body posture and facilitating intraoperative tasks such as adjusting the lighting and delivering surgical instruments.

In the observation group, six-hand operation nursing was employed, involving one circulating nurse, one assistant nurse, and one surgeon. The roles and responsibilities of the nursing staff were as follows:

1. Preoperative Nursing Coordination
  The circulating nurse guided patients through preoperative routine examinations and communicated with the surgeon to understand the therapeutic regimen. Additionally, the nurse provided patients with information about their condition, the purpose of surgical treatment, and potential postoperative complications to alleviate psychological stress, nervousness, and fear. Furthermore, the circulating nurse was responsible for disinfecting the surgical area.
  The assistant nurse was required to be familiar with the surgeon’s operating procedures and preferences. This nurse checked surgical instruments, medications, and materials in an organized manner and assisted the surgeon with disinfection, draping, and anesthesia administration.
2. Intraoperative Nursing Coordination
  The circulating nurse monitored the surgical procedure, adjusted the light source and microscope as needed, and provided surgical items promptly. This nurse also ensured that instruments were properly cleaned, maintained a strict separation between sterile and contaminated areas to prevent cross-infection, and assisted the surgeon in deploying mineral trioxide aggregate.
  The assistant nurse remained seated during surgery, assisting the surgeon by maintaining proper tissue retraction to ensure clear exposure of the affected tooth area. This nurse was also responsible for promptly removing blood and debris from the oral cavity using gauze or suction tubes. Additionally, the assistant nurse protected soft tissues while removing apical tissues, delivered surgical instruments as needed, and cleaned instruments of tissue and blood stains. During suturing, the assistant nurse avoided excessive tension on surrounding soft tissues and assisted the surgeon in cutting the sutures.
3. Postoperative Nursing Coordination
  The circulating nurse guided patients on postoperative care, including the application of cold compresses with ice packs and essential health education.
  Dietary guidelines: Patients were advised to consume fluids on the first postoperative day, followed by semi-solid or soft foods at moderate temperatures on days 2–3. Chewing with the affected tooth was to be avoided, and the use of a straw for drinking was prohibited.
  Oral hygiene and activity restrictions: Brushing teeth, smoking, and strenuous exercise were prohibited on the first postoperative day. Instead, patients were advised to use chlorhexidine mouthwash twice daily for five consecutive days.
  Pain management: Once the anesthesia effect wore off, the circulating nurse guided patients in taking analgesic medication if they experienced intolerable pain.
  Follow-up care: Patients were instructed to attend regular follow-up examinations and have their sutures removed between days 7 and 9 post-surgery.
  The assistant nurse was responsible for resetting the dental chair and workstation, thoroughly disinfecting the contaminated area, and maintaining the surgical microscope. Additionally, the assistant nurse counted, categorized, and organized surgical instruments before sending them to the supply room for sterilization.

## OBSERVATION OF INDICATORS

(1) Operation duration and postoperative cross infection rate.
(2) Therapeutic effects: After nursing, one-week follow-up was completed in both groups to assess therapeutic effects, which were classified into markedly effective (The root tip was completely healed based on X-ray examinations (Siemens, Germany), the radiolucent area and tenderness disappeared, and masticatory function was restored to normal), effective (The root tip was not completely healed according to X-ray examinations, the radiolucent area was partly vanished, the tenderness was relieved, and masticatory function was basically restored) and ineffective (The above criteria were not met).
(3) Pain degree: The pain was evaluated in both groups before nursing and on day 3 after nursing using the Visual Analogue Scale (VAS)^(12)^, with 0, 1–3, 4–6, and 7–10 points for none, mild, moderate, and severe pain, respectively. A higher score indicated more obvious pain.
(4) Inflammatory factors: Fasting venous blood (3 mL) was harvested from patients in both groups before nursing and on day 3 after nursing, followed by centrifugation. Next, the supernatant was collected for measurement of tumor necrosis factor-α (TNF-α) and interleukin-6 (IL-6) levels *via* enzyme-linked immunosorbent assay according to the kit’s instructions (Thermo Fisher Scientific, USA).
(5) Satisfaction with nursing: After the end of nursing, the Newcastle Satisfaction with Nursing Scale (NSNS) was employed to assess the satisfaction with nursing in the two groups^(13)^, which was composed of 19 items, with 1–5 point(s) for each item and a total score of 95 points. A total score of 19–38 points, 39–57 points, 58–76 points and 77–95 points indicated extremely dissatisfied, dissatisfied, basically satisfied and satisfied, respectively.

### Statistical Analysis

SPSS 23.0 software (IBM Inc., USA) was utilized for statistical analysis. Measurement data were expressed as mean ± standard deviation (‾*x* ± *s*) and examined by the *t*-test. Count data were described as frequency and rate (%) and subjected to the χ^2^ test. P < 0.05 denoted that the difference was of statistical significance.

### Ethical Aspects Subsection

This study has received ethical approval from the hospital, and all patients signed the consent form (approval No. JSAM2022-051).

## RESULTS

### Operation Duration and Cross Infection Rate

The operation duration was significantly shorter in the observation group than that in the control group (t = 9.113, P < 0.001). The cross infection rate declined in the observation group compared with that in the control group (χ^2^ = 4.464, P = 0.035) (Table 1).

**Table 1:** Operation duration and cross infection rate - Nanjing, Jiangsu Province, China, 2022.

Group	Operation duration (min)	Cross infection rate	
		Infected	Uninfected
Control (n = 45)	136.58 ± 20.10	6 (13.33)	39 (86.67)
Observation (n = 45)	105.23 ± 11.34	0 (0.00)	45 (100.00)
t/χ^2^	9.113	4.464	
P	<0.001	0.035	

Measurement data were expressed as mean ± standard deviation (‾*x* ± *s*) and examined by the *t*-test. Count data were described as frequency and rate (%) and subjected to the χ^2^ test.

### Vas Scores

Before nursing, the two groups had similar VAS scores (P = 0.875). The VAS score significantly dropped in the observation group on day 3 after nursing in comparison to that in the control group (P < 0.001) (Table 2).

**Table 2:** VAS scores (‾*x* ± *s*, point) - Nanjing, Jiangsu Province, China, 2022.

Group	Before nursing	On day 3 after nursing	t	P
Control (n = 45)	4.86 ± 1.14	2.31 ± 0.56	13.468	<0.001
Observation (n = 45)	4.90 ± 1.26	1.75 ± 0.44	15.833	<0.001
t	0.158	5.275		
P	0.875	<0.001		

### Therapeutic Effects

In comparison with the control group, the observation group had a significantly higher overall response rate (χ^2^ = 4.444, P = 0.035) (Table 3).

**Table 3:** Therapeutic effects [n (%)] - Nanjing, Jiangsu Province, China, 2022.

Group	Markedly effective	Effective	Ineffective	Overall response rate
Control (n = 45)	20 (44.44)	17 (37.78)	8 (17.78)	37 (82.22)
Observation (n = 45)	32 (71.11)	12 (26.67)	1 (2.22)	44 (97.78)
χ^2^				4.444
P				0.035

Count data were described as frequency and rate (%) and subjected to the χ^2^ test.

### Inflammatory Factors

Before nursing, no differences of statistical significance were found in the levels of TNF-α and IL-6 between the two groups (P = 0.922, 0.582). On day 3 after nursing, the levels of TNF-α and IL-6 decreased in the two groups compared with those before nursing, and they were significantly lower in the observation group than those in the control group (P < 0.001) (Table 4).

**Table 4:** Levels of inflammatory factors (‾*x* ± *s*, ng/mL) - Nanjing, Jiangsu Province, China, 2022.

Group	TNF-α		IL-6	
	Before nursing	On day 3 after nursing	Before nursing	On day 3 after nursing
Control (n = 45)	33.76 ± 3.05	23.45 ± 1.64	22.94 ± 1.35	15.94 ± 2.13
Observation (n = 45)	33.83 ± 3.67	18.34 ± 1.12	23.10 ± 1.40	10.25 ± 2.67
t	0.098	17.261	0.552	11.175
P	0.922	<0.001	0.582	<0.001

Measurement data were expressed as mean ± standard deviation (‾*x* ± *s*) and examined by the *t*-test. Satisfaction with nursing.

The observation group had significantly higher overall satisfaction with nursing than that of the control group (χ^2^ = 9.680, P = 0.002) (Table 5).

**Table 5:** Satisfaction with nursing [n (%)] - Nanjing, Jiangsu Province, China, 2022.

Group	Satisfied	Basically satisfied	Dissatisfied	Extremely dissatisfied	Total satisfaction
Control (n = 45)	20 (44.44)	12 (26.67)	10 (22.22)	3 (6.67)	32 (71.11)
Observation (n = 45)	34 (75.56)	9 (20.00)	2 (4.44)	0 (0.00)	43 (95.56)
χ^2^					9.680
P					0.002

Total satisfaction = satisfied + basically satisfied. Count data were described as frequency and rate (%) and subjected to the χ^2^ test.

## DISCUSSION

In the present study, the observation group demonstrated a shorter operation duration, a lower cross-infection rate, a higher overall response rate, and a lower VAS score on day 3 post-nursing compared to the control group. These findings suggest that six-hand operation nursing effectively reduces operation time, controls infection rates, enhances therapeutic outcomes, and alleviates postoperative pain. Several factors may contribute to these advantages.

First, the inclusion of an additional circulating nurse significantly reduces operation duration and improves overall efficiency. Second, in conventional nursing cooperation, the assistant nurse is responsible for preparing medical instruments and materials, thereby increasing the risk of cross-infection. In contrast, six-hand operation nursing assigns the circulating nurse to manage the sterile area while the assistant nurse assists the surgeon in the contaminated area, effectively minimizing the risk of cross-infection^(14)^. Third, providing patients with preoperative education regarding their condition can help alleviate psychological distress and encourage active cooperation during treatment. During surgery, the assistant nurse supports the surgeon in reducing intraoperative traction, thereby protecting soft tissues. Postoperatively, the assistant nurse ensures that patients receive appropriate guidance on analgesic medication. These comprehensive nursing interventions contribute to pain relief and facilitate postoperative recovery^(15,16)^.

Therefore, six-hand operation nursing demonstrates clear advantages over conventional nursing in microscopic apical surgery. By delegating specific responsibilities, medical personnel can ensure smooth surgical procedures while delivering high-quality, efficient nursing care, ultimately improving therapeutic outcomes.

According to studies by Galler et al.^(17)^ and Zaky et al.^(18)^, inflammation plays a significant role in the development and progression of oral diseases. Severe inflammatory responses promote bacterial proliferation in the root canal, allowing pathogens to invade the peri-root bone through the apical foramen and subsequently damage periapical tissues. In this study, TNF-α and IL-6 levels decreased in both groups on day 3 post-nursing compared to baseline levels. However, these inflammatory markers were significantly lower in the observation group than in the control group, suggesting that six-hand operation nursing also contributes to mitigating inflammatory responses, likely due to improved postoperative infection control.

High-quality nursing services foster a harmonious doctor-patient relationship and enhance patient satisfaction with medical care. In this study, nursing satisfaction was significantly higher in the observation group than in the control group. This may be attributed to the fact that, in conventional nursing, medical staff experience high workloads and pressure, whereas the presence of an additional circulating nurse in six-hand operation nursing allows for a more balanced workload distribution. This, in turn, facilitates more comprehensive patient care and reduces discomfort during treatment^(19,20)^. Overall, six-hand operation nursing integrates humanistic care principles, effectively alleviating patients’ psychological distress and significantly improving their satisfaction with nursing services.

### Limitations

Despite these promising findings, this study has certain limitations. First, the sample size was relatively small. Second, the follow-up period was not sufficiently long. Ongoing research in our group aims to address these limitations by incorporating a larger sample size and extending the follow-up duration.

## CONCLUSION

In conclusion, six-hand operation nursing in microscopic apical surgery is beneficial in reducing operation duration, lowering infection rates, alleviating postoperative pain, and enhancing therapeutic outcomes. Additionally, this nursing model helps mitigate inflammatory responses and significantly improves patient satisfaction with nursing care.
