# Supplementary material for: Effect of six hand operation nursing applied in microscopic apical surgery
Source: Rev Esc Enferm USP. 2025 May 9;59:e20240383. doi: 10.1590/1980-220X-REEUSP-2024-0383en (PMC12068243; doi:10.1590/1980-220X-REEUSP-2024-0383en)
Supplement: Translation

# Translation: Efecto de la enfermería de operación a seis manos aplicada en cirugía apical microscópica

**DOI:** 10.1590/1980-220X-REEUSP-2024-0383es

**Authors:** Yahua Liu, Wei Zhang, Su Jiang, Jie Zhang, Li Dai

**Affiliations:** 1 Nanjing University, Medical School, Institute of Stomatology, Nanjing Stomatological Hospital, Department of Endodontics, 210000, Nanjing, China.

**Keywords:** Tejido Periapical, Infecciones, Microscopía, Enfermería, Cirugía General

## Abstract

**Objetivo:**

Nuestro objetivo fue investigar el efecto de la enfermería en seis operaciones manuales aplicadas en cirugía apical microscópica.

**Método:**

Los sujetos (90 en total) fueron pacientes reclutados que se sometieron a cirugía apical microscópica en nuestro hospital entre marzo de 2022 y marzo de 2024. Los participantes se asignaron a un grupo control (n = 45) y a un grupo observación (n = 45) mediante una tabla de números aleatorios.

**Resultados:**

La tasa de respuesta general fue mayor en el grupo observación que en el grupo control (P < 0,05). Los niveles del factor de necrosis tumoral-α y de la interleucina-6, tres días después de la enfermería, disminuyeron en ambos grupos en comparación con los niveles previos a la enfermería, y fueron más bajos en el grupo observación que en el grupo control (P < 0,05). La satisfacción general con la enfermería aumentó en el grupo observación con relación al grupo control (P < 0,05).

**Conclusión:**

La enfermería en seis operaciones manuales aplicadas en cirugía apical microscópica puede disminuir la duración de la operación y reducir el riesgo de infecciones.

## INTRODUCCIÓN

La periodontitis tiene una tasa de prevalencia que aumenta gradualmente y afecta de forma significativa a la vida diaria de las personas^(1)^. En la actualidad, la endodoncia es el principal tratamiento para los pacientes con periodontitis que no responden a los enfoques conservadores. Aunque tanto la endodoncia como el retratamiento han mostrado avances considerables en cuanto a eficacia terapéutica, en algunos pacientes se siguen observando lesiones apicales residuales en los dientes afectados, lo que deteriora gravemente su calidad de vida^(2)^.

En la mayoría de los casos, las lesiones periapicales se tratan mediante cirugía apical, cuyo objetivo es eliminar las infecciones del tejido periapical y promover al mismo tiempo la regeneración tisular y una rápida cicatrización^(3)^. Este procedimiento suele realizarse en un entorno ambulatorio, donde mantener unas condiciones asépticas puede resultar complicado. Además, una carga bacteriana significativa en la saliva de los pacientes supone un riesgo de contaminación del instrumental quirúrgico y de las manos del personal médico, lo que puede dar lugar a infecciones cruzadas y dificultar la recuperación postoperatoria^(4)^. Por lo tanto, el control eficaz de las infecciones durante la cirugía apical es de vital importancia.

La cirugía apical es un método crucial para eliminar los tejidos infectados de la punta de la raíz y facilitar la cicatrización de los tejidos. En los últimos años, la tasa de éxito de este procedimiento ha mejorado significativamente con la incorporación de microscopios quirúrgicos^(5,6)^. Las técnicas microscópicas proporcionan un campo de visión quirúrgico mejorado, lo que permite una identificación precisa de las estructuras fisiológicas periapicales. Sin embargo, la cirugía microscópica impone grandes exigencias tanto a los cirujanos como al personal de enfermería. Es esencial seguir estrictamente las técnicas estériles, y las maniobras quirúrgicas deben reducirse al mínimo en la medida de lo posible^(7)^.

En los cuidados de enfermería convencionales, un solo médico suele colaborar con una enfermera. Sin embargo, esta práctica estándar no satisface las elevadas exigencias técnicas y procedimentales de la cirugía microscópica^(8,9)^. La eficacia de la colaboración perioperatoria de enfermería en los procedimientos microscópicos está directamente relacionada tanto con el riesgo de infección cruzada como con los resultados quirúrgicos generales^(10)^. Por lo tanto, una colaboración eficaz desempeña un papel crucial para garantizar una rápida recuperación postoperatoria.

Para mejorar la calidad de la atención médica, se ha introducido en los equipos quirúrgicos una enfermera circulante -responsable de mantener una transición controlada entre las zonas contaminadas y no contaminadas-, lo que ha dado lugar al concepto de enfermería en operaciones a seis manos^(11)^. Se ha demostrado que este modelo de enfermería, que requiere personal médico experimentado, reduce la duración tanto del diagnóstico como del tratamiento, por lo que es muy preferido por los pacientes^(12)^.

En la actualidad, la investigación sobre la eficacia clínica de este modelo de enfermería en la cirugía apical microscópica sigue siendo limitada, sobre todo en lo que respecta a su repercusión en el control de las infecciones. Dada esta laguna de conocimientos, en este estudio se incluyó a 90 pacientes sometidos a cirugía apical microscópica en nuestro hospital durante los dos últimos años para evaluar la eficacia de la enfermería en la operación a seis manos. El objetivo de los resultados es informar sobre futuros protocolos de enfermería y mejorar las estrategias de cuidados perioperatorios.

## MÉTODO

### Sujetos

Los 90 pacientes sometidos a cirugía apical microscópica en nuestro hospital entre marzo de 2022 y marzo de 2024 se dividieron en un grupo de control (n = 45) y un grupo de observación (n = 45) mediante una tabla de números aleatorios. El grupo de control estaba compuesto por 25 varones y 20 mujeres de edades comprendidas entre 22 y 51 años, con una media de (33,25 ± 3,41) años. El diámetro de la lesión del diente enfermo era de 7–13 mm, con una media de (8,45 ± 1,20) mm. El índice de masa corporal era de 20–24 ^kg/m2^, con una media de (22,36 ± 1,47) ^kg/m2^. En el grupo de observación había 23 varones y 22 mujeres de edades comprendidas entre 23 y 51 años, con una media de (33,86 ± 3,09) años. El diámetro de la lesión del diente enfermo era de 7–14 mm, con una media de (8,64 ± 1,17) mm. El índice de masa corporal oscilaba entre 21 kg/m2^y^ 24 ^kg/m2^, con una media de (22,53 ± 1,37) ^kg/m2^. Los datos generales estaban bien equilibrados entre los dos grupos (P > 0,05).

### Subsección de Aspectos Éticos

Este estudio ha recibido la aprobación ética del hospital, y todos los pacientes firmaron el formulario de consentimiento (aprobación nº JSAM2022–051).

## CRITERIOS DE INCLUSIÓN Y EXCLUSIÓN

Se emplearon los siguientes criterios de inclusión 1) pacientes con fracaso en el primer tratamiento de conducto y que cumplían las indicaciones de reoperación, 2) aquellos con punta radicular no cerrada según las imágenes radiográficas, 3) aquellos con función de coagulación normal, y 4) aquellos que habían firmado voluntariamente el formulario de consentimiento informado.

Los criterios de exclusión fueron: 1) pacientes con otras enfermedades infecciosas, 2) aquellos con fracturas radiculares en el diente afectado, 3) aquellos con enfermedades metabólicas, 4) aquellos con lesiones graves de la pulpa dental, 5) aquellos con perforación lateral o fisura longitudinal del conducto radicular, 6) aquellos con lesiones orgánicas múltiples, 7) aquellos con malformación del desarrollo radicular, u 8) aquellos con trastornos mentales.

## MÉTODOS

En el grupo de control se aplicaron cuidados de enfermería convencionales, en los que participaron una enfermera auxiliar y un cirujano. En concreto, la auxiliar de enfermería asistió al cirujano durante la intervención ayudando a los pacientes a ajustar su postura corporal y facilitando tareas intraoperatorias como el ajuste de la iluminación y la entrega del instrumental quirúrgico.

En el grupo de observación se empleó la enfermería de operaciones a seis manos, con la participación de una enfermera circulante, una enfermera auxiliar y un cirujano. Las funciones y responsabilidades del personal de enfermería eran las siguientes:

1. Coordinación de enfermería preoperatoria
  La enfermera circulante guiaba a los pacientes a través de los exámenes rutinarios preoperatorios y se comunicaba con el cirujano para comprender el régimen terapéutico. Además, la enfermera proporcionaba a los pacientes información sobre su estado, la finalidad del tratamiento quirúrgico y las posibles complicaciones postoperatorias para aliviar el estrés psicológico, el nerviosismo y el miedo. Además, la enfermera circulante se encargaba de desinfectar la zona quirúrgica.
  La enfermera auxiliar debía conocer los procedimientos operativos y las preferencias del cirujano. Esta enfermera revisaba el instrumental quirúrgico, la medicación y el material de forma organizada y ayudaba al cirujano con la desinfección, el vendaje y la administración de la anestesia.
2. Coordinación de enfermería intraoperatoria
  La enfermera circulante monitoreaba el procedimiento quirúrgico, ajustaba la fuente de luz y el microscopio según fuera necesario y proporcionaba los artículos quirúrgicos con prontitud. Esta enfermera también se aseguró de que los instrumentos se limpiaran adecuadamente, mantuvo una separación estricta entre las zonas estériles y contaminadas para evitar infecciones cruzadas y ayudó al cirujano a desplegar el agregado de trióxido mineral.
  La auxiliar de enfermería permaneció sentada durante la intervención y ayudó al cirujano manteniendo una retracción adecuada de los tejidos para garantizar una exposición clara de la zona del diente afectado. Esta enfermera también se encargó de retirar rápidamente la sangre y los restos de la cavidad oral utilizando gasas o tubos de succión. Además, la auxiliar de enfermería protegía los tejidos blandos mientras retiraba los tejidos apicales, entregaba el instrumental quirúrgico según fuera necesario y limpiaba el instrumental de manchas de tejido y sangre. Durante la sutura, la auxiliar de enfermería evitaba una tensión excesiva en los tejidos blandos circundantes y ayudaba al cirujano a cortar las suturas.
3. Coordinación de enfermería postoperatoria
  La enfermera circulante orientó a los pacientes sobre los cuidados postoperatorios, incluida la aplicación de compresas frías con bolsas de hielo y la educación sanitaria esencial.
  Pautas dietéticas: Se aconsejó a los pacientes que consumieran líquidos el primer día del postoperatorio, seguidos de alimentos semisólidos o blandos a temperaturas moderadas los días 2 y 3. La masticación con el diente afectado debía realizarse en la boca. Se evitó masticar con el diente afectado y se prohibió el uso de tubos para beber.
  Higiene bucal y restricciones de actividad: Se prohibió cepillarse los dientes, fumar y hacer ejercicio intenso el primer día postoperatorio. En su lugar, se aconsejó a los pacientes que se enjuagaran la boca con clorhexidina dos veces al día durante cinco días consecutivos.
  Tratamiento del dolor: Una vez pasado el efecto de la anestesia, la enfermera circulante orientó a los pacientes para que tomaran medicación analgésica si experimentaban un dolor intolerable.
  Seguimiento: Se indicó a las pacientes que acudieran a las revisiones periódicas de seguimiento y que se les retiraran las suturas entre los días 7 y 9 del postoperatorio.
  La auxiliar de enfermería se encargó de reajustar el sillón dental y la estación de trabajo, desinfectar a fondo la zona contaminada y mantener el microscopio quirúrgico. Además, la auxiliar de enfermería contaba, clasificaba y organizaba el instrumental quirúrgico antes de enviarlo a la sala de suministros para su esterilización.

## OBSERVACIÓN DE INDICADORES

(1) Duración de la operación y tasa de infección cruzada postoperatoria.
(2) Efectos terapéuticos: Tras la intervención, se realizó un seguimiento de una semana en ambos grupos para evaluar los efectos terapéuticos, que se clasificaron en notablemente eficaces (la punta de la raíz estaba completamente curada según los exámenes radiográficos (Siemens, Alemania), la zona radiotransparente y la sensibilidad habían desaparecido, y la función masticatoria había vuelto a la normalidad), eficaces (la punta de la raíz no estaba completamente curada según los exámenes radiográficos, la zona radiotransparente había desaparecido parcialmente, la sensibilidad se había aliviado, y la función masticatoria había vuelto básicamente a la normalidad) e ineficaces (no se cumplían los criterios anteriores).
(3) Grado de dolor: El dolor se evaluó en ambos grupos antes de los cuidados de enfermería y en el día 3 después de los cuidados de enfermería mediante la escala visual analógica (EVA)^(12)^, con 0, 1–3, 4–6 y 7–10 puntos para dolor nulo, leve, moderado e intenso, respectivamente. Una puntuación más alta indicaba un dolor más evidente.
(4) Factores inflamatorios: Se extrajo sangre venosa en ayunas (3 mL) de los pacientes de ambos grupos antes de los cuidados de enfermería y el día 3 después de los cuidados de enfermería, seguida de centrifugación. A continuación, se recolectó el sobrenadante para medir los niveles de factor de necrosis tumoral-α (TNF-α) e interleucina-6 (IL-6) *mediante* un ensayo inmunoenzimático de acuerdo con las instrucciones del kit (Thermo Fisher Scientific, EE.UU.).
(5) Satisfacción con los cuidados de enfermería: Tras el final de la enfermería, se empleó la Escala de Satisfacción con la Enfermería de Newcastle (Newcastle Satisfaction with Nursing Scale - NSNS) para evaluar la satisfacción con la enfermería en los dos grupos^(13)^, que estaba compuesta por 19 ítems, con 1–5 punto(s) para cada ítem y una puntuación total de 95 puntos. Una puntuación total de 19–38 puntos, 39–57 puntos, 58–76 puntos y 77–95 puntos indicaba extremadamente insatisfecho, insatisfecho, básicamente satisfecho y satisfecho, respectivamente.

### Análisis Estadístico

Para el análisis estadístico se utilizó el programa SPSS 23.0 (IBM Inc., EE.UU.). Los datos de medición se expresaron como media ± desviación estándar (`x ± s) y se examinaron mediante la prueba *t*. Los datos de recuento se describieron como frecuencia y tasa (%) y se sometieron a la prueba de la χ^2^. P < 0,05 denotaba que la diferencia tenía significación estadística.

### Subsección de Aspectos Éticos

Este estudio ha recibido la aprobación ética del hospital, y todos los pacientes firmaron el formulario de consentimiento (aprobación nº JSAM2022–051).

## RESULTADOS

### Duración de la Operación y Tasa de Infección Cruzada

La duración de la operación fue significativamente menor en el grupo de observación que en el grupo de control (t = 9,113; P < 0,001). La tasa de infección cruzada disminuyó en el grupo de observación en comparación con la del grupo de control (χ^2^ = 4,464; P = 0,035) (Tabla 1).

**Tabla 1:** Duración de la operación y tasa de infección cruzada - Nanjing, provincia de Jiangsu, China, 2022.

Grupo	Duración de la operación (min)	Tasa de infección cruzada	
		Infectados	No infectados
Control (n = 45)	136.58 ± 20.10	6 (13.33)	39 (86.67)
Observación (n = 45)	105.23 ± 11.34	0 (0.00)	45 (100.00)
t/χ^2^	9.113	4.464	
P	<0.001	0.035	

Los datos de medición se expresaron como media ± desviación estándar (`x ± s) y se examinaron mediante la prueba *t*. Los datos de recuento se describieron como frecuencia y tasa (%) y se sometieron a la prueba de χ^2^.

### Puntuaciones de la Eva

Antes de los cuidados de enfermería, los dos grupos tenían puntuaciones de la EVA similares (P = 0,875). La puntuación EVA disminuyó significativamente en el grupo de observación el tercer día después de los cuidados de enfermería en comparación con la del grupo de control (P < 0,001) (Tabla 2).

**Tabla 2:** Puntuaciones EVA (`x ± s, punto) - Nanjing, provincia de Jiangsu, China, 2022.

Grupo	Antes de los cuidados de enfermería	En el dia tres después de los cuidados	t	P
Control (n = 45)	4.86 ± 1.14	2.31 ± 0.56	13.468	<0.001
Observación (n = 45)	4.90 ± 1.26	1.75 ± 0.44	15.833	<0.001
t	0.158	5.275		
P	0.875	<0.001		

### Efectos Terapéuticos

En comparación con el grupo de control, el grupo de observación tuvo una tasa de respuesta global significativamente mayor (χ^2^ = 4,444, P = 0,035) (Tabla 3).

**Tabla 3:** Efectos terapéuticos [n (%)] - Nanjing, provincia de Jiangsu, China, 2022.

Grupos	Marcadamente eficaz	Efectiva	Inefectiva	Tasa de respuesta total
Control (n = 45)	20 (44.44)	17 (37.78)	8 (17.78)	37 (82.22)
Observación (n = 45)	32 (71.11)	12 (26.67)	1 (2.22)	44 (97.78)
χ^2^				4.444
P				0.035

Los datos de recuento se describieron como frecuencia y tasa (%) y se sometieron a la prueba de la χ^2^.

### Factores Inflamatorios

Antes de los cuidados de enfermería, no se encontraron diferencias con significación estadística en los niveles de TNF-α e IL-6 entre los dos grupos (P = 0,922, 0,582). El tercer día después de la lactancia, los niveles de TNF-α e IL-6 disminuyeron en los dos grupos en comparación con los de antes de la lactancia, y fueron significativamente más bajos en el grupo de observación que en el grupo de control (P < 0,001) (Tabla 4).

**Tabla 4:** Niveles de factores inflamatorios (`x ± s, ng/mL) - Nanjing, provincia de Jiangsu, China, 2022.

Grupo	TNF-α		IL-6	
	Antes de los cuidados de enfermería	En el dia tres después de los cuidados	Antes de los cuidados de enfermería	En el dia tres después de los cuidados
Control (n = 45)	33.76 ± 3.05	23.45 ± 1.64	22.94 ± 1.35	15.94 ± 2.13
Observación (n = 45)	33.83 ± 3.67	18.34 ± 1.12	23.10 ± 1.40	10.25 ± 2.67
t	0.098	17.261	0.552	11.175
P	0.922	<0.001	0.582	<0.001

Los datos de las mediciones se expresaron como media ± desviación estándar (`x ± s) y se examinaron mediante la prueba *t*.

### Satisfacción Con Los Cuidados De Enfermería

El grupo de observación tuvo una satisfacción global con los cuidados de enfermería significativamente mayor que la del grupo de control (χ^2^ = 9,680, P = 0,002) (Tabla 5).

**Tabla 5:** Satisfacción con los cuidados de enfermería [n (%)] - Nanjing, provincia de Jiangsu, China, 2022.

Grupo	Satisfechos	Básicamente satisfecho	Insatisfecho	Extremamente insatisfecho	Satisfacción total
Control (n = 45)	20 (44.44)	12 (26.67)	10 (22.22)	3 (6.67)	32 (71.11)
Observación (n = 45)	34 (75.56)	9 (20.00)	2 (4.44)	0 (0.00)	43 (95.56)
χ^2^					9.680
P					0.002

Satisfacción total = satisfecho + básicamente satisfecho. Los datos de recuento se describieron como frecuencia y tasa (%) y se sometieron a la prueba de la χ^2^.

## DISCUSIÓN

En el presente estudio, el grupo de observación demostró una menor duración de la operación, una menor tasa de infección cruzada, una mayor tasa de respuesta global y una menor puntuación de la EVA el tercer día después de los cuidados de enfermería en comparación con el grupo de control. Estos resultados sugieren que la enfermería a seis manos reduce eficazmente el tiempo de la operación, controla las tasas de infección, mejora los resultados terapéuticos y alivia el dolor postoperatorio. Varios factores pueden contribuir a estas ventajas.

En primer lugar, la inclusión de una enfermera circulante adicional reduce significativamente la duración de la operación y mejora la eficiencia global. En segundo lugar, en la cooperación enfermera convencional, la enfermera auxiliar se encarga de preparar el instrumental y el material médico, lo que aumenta el riesgo de infecciones cruzadas. En cambio, la enfermería de operaciones a seis manos asigna a la enfermera circulante la gestión de la zona estéril, mientras que la enfermera auxiliar ayuda al cirujano en la zona contaminada, lo que minimiza eficazmente el riesgo de infección cruzada^(14)^. En tercer lugar, proporcionar a los pacientes educación preoperatoria sobre su estado puede ayudar a aliviar la angustia psicológica y fomentar la cooperación activa durante el tratamiento. Durante la intervención, el auxiliar de enfermería ayuda al cirujano a reducir la tracción intraoperatoria, protegiendo así los tejidos blandos. En el postoperatorio, el auxiliar de enfermería se asegura de que los pacientes reciban una orientación adecuada sobre la medicación analgésica. Estas intervenciones integrales de enfermería contribuyen a aliviar el dolor y facilitan la recuperación postoperatoria^(15,16)^.

Por lo tanto, la enfermería a seis manos demuestra claras ventajas sobre la enfermería convencional en la cirugía apical microscópica. Al delegar responsabilidades específicas, el personal médico puede garantizar la fluidez de los procedimientos quirúrgicos al tiempo que presta cuidados de enfermería eficientes y de alta calidad, lo que en última instancia mejora los resultados terapéuticos.

Según los estudios de Galler et al.^(17)^ y Zaky et al.^(18)^, la inflamación desempeña un papel importante en el desarrollo y la progresión de las enfermedades bucodentales. Las respuestas inflamatorias graves promueven la proliferación bacteriana en el conducto radicular, lo que permite a los patógenos invadir el hueso perirradicular a través del foramen apical y dañar posteriormente los tejidos periapicales. En este estudio, los niveles de TNF-α e IL-6 disminuyeron en ambos grupos en el día 3 posenfermería en comparación con los niveles basales. Sin embargo, estos marcadores inflamatorios fueron significativamente más bajos en el grupo de observación que en el grupo de control, lo que sugiere que la enfermería en la operación a seis manos también contribuye a mitigar las respuestas inflamatorias, probablemente debido a un mejor control de la infección postoperatoria.

Los servicios de enfermería de alta calidad fomentan una relación médico-paciente armoniosa y mejoran la satisfacción del paciente con la atención médica. En este estudio, la satisfacción del personal de enfermería fue significativamente mayor en el grupo de observación que en el grupo de control. Esto puede atribuirse al hecho de que, en la enfermería convencional, el personal médico experimenta una gran carga de trabajo y presión, mientras que la presencia de una enfermera circulante adicional en la enfermería de operaciones a seis manos permite una distribución más equilibrada de la carga de trabajo. Esto, a su vez, facilita una atención más completa al paciente y reduce las molestias durante el tratamiento^(19,20)^. En general, la enfermería a seis manos integra los principios de los cuidados humanistas, aliviando eficazmente el malestar psicológico de los pacientes y mejorando significativamente su satisfacción con los servicios de enfermería.

### Limitaciones

A pesar de estos resultados prometedores, este estudio tiene ciertas limitaciones. En primer lugar, el tamaño de la muestra era relativamente pequeño. En segundo lugar, el periodo de seguimiento no fue suficientemente largo. Las investigaciones en curso de nuestro grupo pretenden abordar estas limitaciones incorporando una muestra de mayor tamaño y ampliando la duración del seguimiento.

## CONCLUSIÓN

En conclusión, la enfermería a seis manos en la cirugía apical microscópica es beneficiosa para reducir la duración de la operación, disminuir las tasas de infección, aliviar el dolor postoperatorio y mejorar los resultados terapéuticos. Además, este modelo de enfermería ayuda a mitigar las respuestas inflamatorias y mejora significativamente la satisfacción del paciente con los cuidados de enfermería.
